# Teacher Stroke as a Positive Interpersonal Behavior on English as a Foreign Language Learners' Success and Enthusiasm

**DOI:** 10.3389/fpsyg.2021.761658

**Published:** 2021-10-20

**Authors:** Zhe Song

**Affiliations:** School of Economics and Management, Yantai University, Yantai, China

**Keywords:** positive interpersonal behavior, students' enthusiasm, students' success, teacher's stroke, EFL

## Abstract

In the context of language learning, teacher-student interactions are regarded as pivotal for their impact on the education of learners and their academic journey. To help learners succeed, the teachers may employ various ways to develop this interpersonal relationship, one of which is teacher stroke which is also called teacher praise. Interpersonal skills, societal encouragement, and stroking manners, respect, consideration, or reactions that an individual provides for others can uphold such a connection and can affect the enthusiasm of the students. To this end, this study makes an effort to review the prominence of teacher stroke in the process of learning in the classroom and illustrate their relationship and their impact on the enthusiasm and success of the learners. Furthermore, this study contributes to the body of dominant literature and suggestions and recommendations have been presented for the language teaching stakeholders in the educational setting.

## Introduction

Among a bulk of the elements providing satisfying learning practices for the learners in the context of English as a foreign language (EFL), their success and enthusiasm in the classroom should be taken into account (Syahabuddin et al., 2020). Indeed, the concept of enthusiasm has been viewed as the primary element of the success and achievement of learners in both individual and educational lifetimes (Gopalan et al., 2020). In this domain, it has been frequently scrutinized from diverse directions and perceptions, which consistently confirmed the effect of enthusiasm on the progression of language education (Al-Hoorie, 2017). According to the review of the literature regarding the motivational aspect of language education, it is speculated that a high degree of enthusiasm can be achieved from various resources, namely parents, peers, and educators; however, in the learning route, educators have a noteworthy function to take charge of it (Varga, 2017), since they are viewed as the most significant stakeholders in all the instructive settings who can regulate the degree and superiority of success and interaction capability of learners, particularly in a language framework (Pishghadam et al., 2021). Similarly, constructing a positive association and interaction among learners are the ways to stimulate and encourage them in learning a language. Al-Nasseri (2014) suggested that when the teachers care about the confidence of the learners and nurture their self-trust, their motivation is enhanced. Also, the teacher-student rapport builds the emotional tie of the learner (Allen et al., 2013) and this relationship is worthwhile in language education where educators and learners are in persistent communication, construct more friendly interactions, and are involved in social communications as a way to develop the language ability of the learners (Pishghadam et al., 2019; Sun et al., 2019; Fathi et al., 2021). In conjunction with the verdicts and the activities of the educators, their social, spiritual, and educational aspects are conspicuous in academic circles and language learning (Derakhshan et al., 2020; Derakhshan, 2021). The positive collaboration between the educators and learners offers a suitable instructional setting in line with positive psychology in which the learners appreciate their learning knowledge (MacIntyre et al., 2019; Budzínska and Majchrzak, 2021; Wang et al., 2021).

Moreover, the influence of teacher relational behaviors on the educational commitment, success, and enthusiasm of the learners is the basis of most studies these days (Gao, 2021; Xie and Derakhshan, 2021). Moreover, positive relationships with the educators and learners can support them in dealing with the burdens of the learning process and motivate constructive educational behaviors (Roorda et al., 2011). Such a relationship not only supports the learners to construct the required personal skills, reduces their apprehension, and enhances their enthusiasm, but also helps them to be involved in the education progression (Da Luz, 2015).

In any communication, educators and learners may impact one another either negatively or positively (Pishghadam et al., 2019; Luo et al., 2020). On the one hand, a positive relationship constructively develops into a meaningful basis of provision, develops the enthusiasm of the students, creates a chance to found some compulsory interpersonal abilities (Khajavy et al., 2016), and have an appropriate impression on their enthusiasm and engagement (Van Uden et al., 2014; Martin and Collie, 2019; Derakhshan, 2021). On the other hand, a negative relationship may bring about numerous opposing values such as boredom (Li and Dewaele, 2020; Derakhshan et al., 2021; Kruk, 2021; Li, 2021; Li et al., 2021; Wang and Derakhshan, 2021; Zawodniak et al., 2021).

Besides, students need to be recognized by the teacher in any interaction and get the care of the educator to fulfill their emotional desires (Pishghadam and Khajavy, 2014). One of the main elements that can have a constructive influence on the value of teacher-student relation is stroke, which is a fundamental section of teacher care, referring to the gratitude of the learner educator during rapport (Pishghadam et al., 2015; Derakhshan et al., 2019). The construct of stroke is frequently employed in learning psychology to talk about “teacher feedback” and “teacher praise” (Amini et al., 2019). Similarly, Rajabnejad et al. (2017) acknowledged that the stroke of teachers encourages learners to echo the appropriate manners that are critical for their success and it is stated that teacher stroke is connected to a great level of commitment from the learners (Van Uden et al., 2014; Pishghadam et al., 2021; Zheng, 2021) that consequently leads to the enhancement of the enthusiasm of the learners and higher levels of achievement (Derakhshan et al., 2019; Pishghadam et al., 2021). In the same way, it is proved that educators can enhance the commitment of the learners in a stroke-rich educational situation (Baños et al., 2019).

Even though few studies on stroke have been completed, some systematic reviews have been carried out about the issue, and have piqued the attention of researchers (Irajzad et al., 2017; Noorbakhsh et al., 2018). Nevertheless, based on the information of the researcher, the presentation of a review study on such a positive interpersonal behavior, namely stroke in an EFL context and its functions on the success and enthusiasm of the learners has been unnoticed. Regarding this gap, the present minireview attempts to review the history, definitions, and future guidelines of teacher stroke.

## Teacher Stroke

Positive interactions of the educators with learners can stimulate the ability of the learners and assist them to construct the required relational abilities (Pierson, 2003). These rapports can be inspected by a theory, suggested by Eric Berne, called transactional analysis (TA) theory. This theory is associated with behavior and analysis for individual development and modification (Stewart and Jones, 1987, as cited in Estaji and Rad, 2017). Through TA, the teachers and students are assisted in having creative interactions in the learning settings, and the construct of stroke, as one of the six major mechanisms of TA theory, is labeled as any acts to be done with the attendance of others (Shirai, 2006). Strokes are classified into verbal or nonverbal in which the former refers to a set of locutions assumed as a type of appreciation to another, while the latter range from corporeal touch to a smile, a compliment, or even a signal of endorsement or refusal (Stewart and Joines, 2008). Moreover, Francis and Woodcock (1996, as cited in Irajzad et al., 2017) pinpointed that motivating others may be succeeded in two ways. The first one is positive augmentation that is the act of giving encouraging and constructive strokes to strengthen encouraging manners, and the other one is destructive augmentation that is grounded on providing negative strokes to minimize errors and boost better presentation, which means that enthusiasm is straightforwardly interrelated to stroke.

## Implications and Future Directions

Based on the abovementioned study, since teacher-learner relations bring about a dynamic classroom environment and the success of the learners, educators should be conscious of the issue that stroking should be an indivisible section of their career and can be a supportive way of classroom supervision. So, the educational experts are encouraged to use the applied methods for successful involvement and optimal positive interpersonal behavior, namely strokes that boost the success and enthusiasm of the learners.

A positive relational behavior has concrete implications for the teachers in which they can provide beneficial evidence for teaching. Interpersonal interaction is essential to learner achievement, and building rapport is an operational manner of cooperating with students leading to achievement, engagement, and motivation. Concerning the implication of stroke in learning situations, the educators can be cognizant of the way in which they provide strokes and praises to their learners and, thus, they can upturn the enthusiasm of the learners. Learners can accomplish higher degrees of presentation. Furthermore, teacher stroke has been revealed as offering encouragement, self-assurance, and worthy teacher-learner interactions. It is assumed that in educational psychology, teacher care, in general, and teacher stroke, in particular, are an indispensable foundation of care for influential student presentation and are noteworthy tactics in involving learners in the route of learning and admiring learners in the classroom that promote language motivation of the learner and manners (Derakhshan et al., 2019).

In this study, the supervisors imply that they should boost stroking in the institutional atmosphere to help develop the efficacy of their instructors. In fact, due to the critical role of stroking as an integral constituent of the careers of the teachers, it can help them enhance rapport with their learners, coworkers, and administrators. It has been shown that teachers rich in stroking are more consistent in their interactions and consequently their learners are inclined to attain their learning objectives more efficaciously. Similarly, it must be noted that strokes as a foundation of teacher care must be emphasized by supervisors and implemented in learning policy.

Similarly, materials developers and syllabus designers could embrace some tasks and activities stimulating communication and interaction including some types of strokes (verbal or nonverbal), so that both the teachers and students could get a better sense of language and as a result a higher nous of enthusiasm; accordingly, they could better achieve their purposes, which are in line with the scholastic determinations of the resources and textbooks.

Furthermore, a study of the stroke construct, which was underlined in this study, can be carried out by using a mixed-methods research design to explore if this construct has any association with EFL teachers and the success and enthusiasm of their students. Qualitative research is also recommended to inspect this kind of stroke, which have a more considerable effect on the achievement of the EFL learners.

## Author Contributions

ZS confirms being the sole author of this manuscript wrote up the draft and got it ready for submission to Frontiers in Psychology.

## Funding

This review was supported by Social Science Planning Project of Shandong Province, Research on invisible government in the field of administrative examination and approval in the context of interest game (grant no. JG16J08).

## Conflict of Interest

The author declares that the research was conducted in the absence of any commercial or financial relationships that could be construed as a potential conflict of interest.

## Publisher's Note

All claims expressed in this article are solely those of the authors and do not necessarily represent those of their affiliated organizations, or those of the publisher, the editors and the reviewers. Any product that may be evaluated in this article, or claim that may be made by its manufacturer, is not guaranteed or endorsed by the publisher.
